# Distinct microbial community along the chronic oil pollution continuum of the Persian Gulf converge with oil spill accidents

**DOI:** 10.1038/s41598-021-90735-0

**Published:** 2021-05-31

**Authors:** Maryam Rezaei Somee, Seyed Mohammad Mehdi Dastgheib, Mahmoud Shavandi, Leila Ghanbari Maman, Kaveh Kavousi, Mohammad Ali Amoozegar, Maliheh Mehrshad

**Affiliations:** 1 Extremophiles Laboratory, Department of Microbiology, School of Biology and Center of Excellence in Phylogeny of Living Organisms, College of Science, University of Tehran, Tehran, Iran; 2 Biotechnology and Microbiology Research Group, Research Institute of Petroleum Industry, Tehran, Iran; 3 Laboratory of Complex Biological Systems and Bioinformatics (CBB), Institute of Biochemistry and Biophysics, University of Tehran, Tehran, Iran; 4 Department of Ecology and Genetics, Limnology and Science for Life Laboratory, Uppsala University, Uppsala, Sweden

**Keywords:** Microbial ecology, Water microbiology, Metagenomics

## Abstract

The Persian Gulf, hosting *ca.* 48% of the world’s oil reserves, has been chronically exposed to natural oil seepage. Oil spill studies show a shift in microbial community composition in response to oil pollution; however, the influence of chronic oil exposure on the microbial community remains unknown. We performed genome-resolved comparative analyses of the water and sediment samples along Persian Gulf’s pollution continuum (Strait of Hormuz, Asalouyeh, and Khark Island). Continuous exposure to trace amounts of pollution primed the intrinsic and rare marine oil-degrading microbes such as *Oceanospirillales*, *Flavobacteriales*, *Alteromonadales*, and *Rhodobacterales* to bloom in response to oil pollution in Asalouyeh and Khark samples. Comparative analysis of the Persian Gulf samples with 106 oil-polluted marine samples reveals that the hydrocarbon type, exposure time, and sediment depth are the main determinants of microbial response to pollution. High aliphatic content of the pollution enriched for *Oceanospirillales*, *Alteromonadales,* and *Pseudomonadales* whereas, *Alteromonadales*, *Cellvibrionales*, *Flavobacteriales,* and *Rhodobacterales* dominate polyaromatic polluted samples. In chronic exposure and oil spill events, the community composition converges towards higher dominance of oil-degrading constituents while promoting the division of labor for successful bioremediation.

## Introduction

Exposure to oil and gas derivatives in marine ecosystems rich in oil reservoirs is inevitable due to natural seepage. Intensive industrial oil exploration and transit over the last century have further increased the risk of pollution in these ecosystems^1^. The Persian Gulf is a relatively shallow evaporative basin that hosts more than 48% of the world’s oil reservoirs. The largest recorded oil spill in the Persian Gulf dates back to 1991^2^. However, this ecosystem has been chronically exposed to oil pollution through natural seepage, accidental oil derivatives release from transit tankers or refinery facilities, and discharge of oily wastes and heavy metals from offshore drilling sites.

The two major water inputs of the Persian Gulf include the Indian Ocean surface water entering through the Strait of Hormuz and Arvandrood river freshwater influx from the northwest side. These two streams shape the water circulation of the Persian Gulf in two branches, one along the Iranian coast and the other one along the Arabian coast. The retention time of this semi-enclosed water body is estimated to be around 2–5 years, where the northern part of the Gulf contains the oldest water^3^. The denser and more saline water partially exits the basin through the southern part of the Strait of Hormuz in depth. Therefore, the limited water circulation in the Persian Gulf prolongs the residence time of pollutants in the basin^3^.

While representing low abundances in the pristine marine community, intrinsic oil-degrading taxa bloom in response to oil pollution and play a critical role in the bioremediation process^4^. Because of their vigilance in responding to pollution, they could be considered as microbial indicators of trace oil pollution^5^.

In oil spill accidents, the natural marine microbial community exposed to oil; responds with fluctuating composition as different oil components are gradually degraded^6^. As the input water is carried along the Gulf’s water circulation in the Persian Gulf, it is exposed to varying types of oil derivatives in different locations. Sporadic cultivation efforts have isolated naphthalene degrading (*Shewanella*, *Salegentibacter*, *Halomonas*, *Marinobacter*, *Oceanicola*, *Idiomarina,* and *Thalassospira*)^7^ and crude oil utilizing (*Acinetobacter*, *Halomonas*, *Alcanivorax*, *Marinobacter*, *Microbacterium*, and *Rhodococcus*^8^) bacteria from the Persian Gulf water and sediment samples. A single 16S rRNA amplicon study of the Mangrove forest’s sediment shows a community dominated by *Alteromonadales* (*Marinobacter* and *Idiomarina*) and *Oceanospirillales* (*Alcanivorax* and *Halomonas*) orders from Gammaproteobacteria and genera *Meridianimaribacter* and *Muricauda* from the family *Flavobacteriaceae*^9^. However, the impact of chronic exposure to oil derivatives and recurring pollutions on the microbiome of the Persian Gulf, their oil bioremediation capability, and recovery potential remains largely unknown.

In this study, we aim to (i) explore the dynamics of the input microbial community along the pollution continuum (a gradient from low to high oil contamination) and (ii) understand their metabolic capability for oil bioremediation. To do so, we performed genome-resolved comparative analyses of the Persian Gulf’s water and sediment samples. Additionally, our study aims to compare the microbial community of the Persian Gulf with the publicly available metagenomes of oil-polluted marine water (n = 41) and sediment (n = 65) samples.

## Materials and methods

### Sampling site description, sample collection, and DNA extraction

Three locations in the Persian Gulf (abbreviated as PG) were selected for sampling based on their different level of exposure to oil pollution. The PG receives its major marine water input from the Indian Ocean through the Strait of Hormuz in spring (peaking around May–June), while the Arvandrood river delta in the northwest feeds the Gulf with freshwater input^10^. Sampling locations were selected based on their level of exposure to the oil pollution and sampled in spring and summer 2018 (Supplementary Table S1, sheet 1). Sampling was done once at each location. Water and sediment samples from the Gulf’s input water at the Strait of Hormuz (27.06112 N, 56.24636 E) were collected in May 2018. Along the water circulation current, water and sediment samples close to Asalouyeh county in Bushehr province (27.31767 N, 52.32542 E) and Khark Island (29.13194 N, 50.18105 E) were also collected in May and September 2018, respectively. Asalouyeh hosts a wide variety of natural gas and petrochemical industries, and it’s surrounding water is exposed to aromatic compounds pollution^11^. Khark Island is the most critical oil transportation hub of the Persian Gulf, continuously exposed to oil pollution^12^.

Water and sediment samples were collected using a Niskin bottle and a grab sampling device KC Denmark (Van Veen Grab 2500 cm^2^), respectively. Salinity, pH, dissolved oxygen (DO) concentration, conductivity, and temperature of each sample were measured by HQ40D Portable Multi Meter (HACH). DO concentration was measured in mg/l unit and triplicate. The average value of these triplicate measurements is represented in Supplementary Table S1. For on site DO concentration measurement, we transferred 1 L of the samples collected from 5 m depth to the glass bottle and measured the DO concentration by inserting the probe in water.

Chlorophyll-a concentration of water samples was measured according to the protocol described elsewhere^13^. Total petroleum hydrocarbon (TPH) content of water and sediment samples was measured by GC-FID method^14^. The PAH concentration of water and sediment samples were determined by GC–MS (Agilent 5975C Series, EPA 8270C standard) and HPLC (Agilent 1200 Series, ISO 13877 standard) techniques, respectively^15^. Since the TPH content of the Khark sediment sample was higher than 1 µg/g, the carbon distribution of this sample was also determined using the Simulated Distillation (GC-SimDis) method based on ASTM D2887 standard^16^. Other elements (e.g., Al, As, Ba, Be, Ca, Cl, Co, Cr, Cu, Fe, K, Li, Mg, Mn, Na, Ni, P, Pb, Rb, S, Sr, Ti, U and Zn) of each sample were also detected by ICP-MS (Agilent 4500 Series) (Supplementary Table S1, ionic content).

For each sampling point, 20 L of water samples were collected from 5 m depth. Samples were pre-filtered through 20 µm (Albet DP5891150, Germany), and 5 µm pore-size (Albet DP5895150, Germany) filters (15 cm in diameter). Biomass was finally concentrated on 0.22 µm pore-size cellulose acetate filters (Sartorius 11107-142-N, Germany) using a peristaltic pump. Sediment samples were collected using a grab sampling device. Water filters and sediment samples were stored on dry ice for transfer to the lab.

A standard phenol–chloroform protocol^17^ was used for extracting community DNA from the water samples. Extraction of DNA from sediment samples was carried out using DNeasy PowerMax Soil DNA Extraction Kit (QIAGEN 12988-10, Germany) according to the manufacturer’s instruction. Extracted DNA samples were sequenced using Illumina Novaseq 6000 platform (PE150) (Novogene, Hong Kong).

### Estimation of 16S rDNA abundance by qPCR

*Haloferax volcanii* (IBRC-M 10248) and *Escherichia coli* (IBRC-M 11074) were selected to draw the standard curve as representative of domain archaea and bacteria, respectively. Their genomic DNA was extracted^18^ and the 16S rDNA was amplified using universal primers [21F (TCCGGTTGATCCYGCCGG (Y = C/T)] and 1492R (GGTTACCTTGTTACGACTT) for archaea and 27F (AGAGTTTGATCMTGGCTCAG) and 1492R (GGTTACCTTGTTACGACTT) for bacteria)^19^. The concentration of DNA was measured using Nanodrop (Thermo Nanodrop One/One-C Micro Volume Spectrophotometers). Copy number of double-strand DNA was estimated according to the formula: number of copies per µl = (concentration of PCR product (µl) × 6.022 × 10^23^)/(length of PCR product (bp) × 1 × 10^9^ × 650) in which, 650 is the molecular weight of one base pair in double-strand DNA and 6.022 × 10^23^ is Avogadro number. Domain-specific 16S rRNA primers named 338F (ACTCCTACGGGAGGCAGCAG), 533R (TTACCGCGGCTGCTGGCAC)^20^ and Parch519F (CAGCCGCCGCGGTAA), ARC915R (GTGCTCCCCCGCCAATTCCT)^21^ were selected to detect Bacteria and Archaea respectively. The qPCR was performed using the Power SYBR Green PCR Master Mix (BIOFACT, South Korea) in the MIC real-time PCR system (BioMolecular Systems, Australia). The unknown 16S rRNA copy number of each sample was calculated according to the standard curves (R^2^ value was higher than 99.0% in both curves). The total content of prokaryotes in each sample was calculated using the sum of 16S rDNA copies of bacteria and archaea.

### Reference metagenome collection

For comparative analyses, publicly available metagenomes deposited to sequence read archive (SRA) of the GenBank were screened for the available metagenomic datasets originating from oil-polluted marine water and sediment samples. A total of 41 marine water and 65 marine sediment metagenomic datasets were collected. A detailed description of these metagenomes is shown in Supplementary Table S3. Water samples originated from Norway (Trondheimsfjord, *n* = 17), Deepwater Horizon (Gulf of Mexico, *n* = 13), the northern part of the Gulf of Mexico (dead zone, *n* = 6), and Coal Oil Point of Santa Barbara (*n* = 5). Sediment samples originated from DWH Sediment (Barataria Bay, *n* = *4*5), Municipal Pensacola Beach (USA, *n* = 16), and a hydrothermal vent in the Guaymas Basin (Gulf of California, *n* = 4).

### Ribosomal RNA classification

A subset of 5 million reads was separated from each dataset, and the reads affiliated to ribosomal RNA genes (16S/18S) were detected using SSU-ALIGN^22^. BLAST comparison of putative prokaryotic 16S rRNA sequences against the SILVA reference database (release 132SSUParc) and their taxonomic affiliation was assigned based on their closest hit if the read was ≥ 90 bp at the similarity threshold of ≥ 90.

Non-metric multidimensional scaling (NMDS) analysis of oil-polluted marine water and sediment metagenomic samples worldwide together with the PG samples was performed using the vegan package in Rstudio based on Bray–Curtis dissimilarity of the abundance of unassembled 16S rRNA reads of metagenomes (order-level). The Alpha diversity of samples was also measured using the vegan package in Rstudio based on the Shannon–Wiener index.

### Sequence assembly, binning, and annotation

Paired-end reads of the PG sequenced datasets were interleaved using reformat.sh and quality trimmed by bbduck.sh scripts of BBMap toolkit^23^. All trimmed sequences of each dataset were assembled separately using MEGAHIT (k-mer list 49, 69, 89, 109, 129 and 149)^24^. Only contigs ≥ 1 kb were binned into metagenome assembled genomes (MAGs) based on their different mapping depth and tetranucleotide frequency, using MetaBat2 software^25^. Contamination and completeness of each MAG were evaluated using CheckM and MAGs with completeness above 40% and contamination lower than 5% were considered for further analysis^26^. The taxonomic affiliation of bins was assigned using GTDB-tk^27^. Putative genes were predicted using Prodigal^28^ and preliminarily annotated using Prokka in the metagenomics mood^29^. Predicted protein sequences of each MAG were further annotated using eggNOG-mapper^30^ and PfamScanner^31^.

## Results and discussion

### Persian Gulf water and sediment samples along the oil pollution continuum

**Figure 1:**
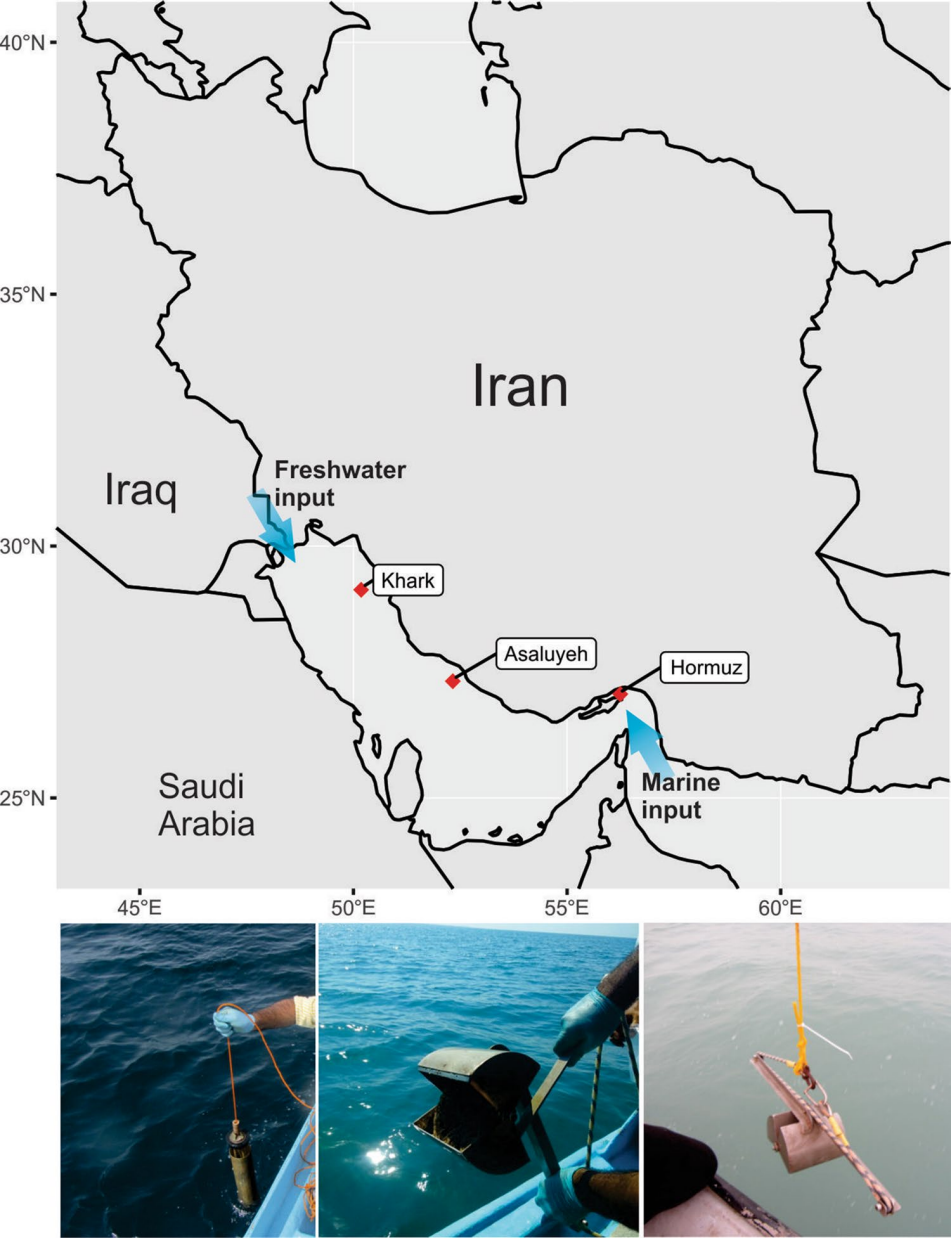
Geographical location of the sampling sites along the pollution continuum of the Persian Gulf. Hormuz sampling point is located close to the Persian Gulf’s marine input water, Asalouyeh sampling point is mainly exposed to aromatic oil contaminants and Khark sampling point is exposed to pollution with different crude oil derivatives. Sampling locations are marked with red squares. Figure was plotted using the open-source maps of the “rnaturalearth” library in R.

Water and sediment samples were collected along the circulation current of the Persian Gulf from Hormuz Island [HW (SAMN12878178) and HS (SAMN12878113)), Asaluyeh area (AW (SAMN12878179) and AS (SAMN12878114)), and Khark Island (KhW (SAMN12878180) and KhS (SAMN12878115)] (Fig. 1). Physicochemical characteristics and Ionic content of the collected samples are presented in the Supplementary Table S1. The GC-FID analyses showed high TPH and polyaromatic hydrocarbon (PAH) concentrations in the Khark sediment (KhS) (Supplementary Table S2). The GC-SimDis analysis showed that C_25_–C_38_ HCs were dominant in the KhS (~ 60%), followed by > C_40_ HCs (~ 14%) (Supplementary Fig. S1). Chrysene, fluoranthene, naphthalene, benzo(a)anthracene and phenanthrene were respectively the most abundant PAHs in KhS. This pollution could originate from oil spillage due to Island airstrikes during the imposed war (1980–1988), sub-sea pipeline failures, and discharge of oily wastewater or ballast water of oil tankers (ongoing for ~ 50 years)^12^. The TPH of other water and sediment samples was below the detection limit of our method (< 50 µg/L and 1 µg/g, respectively). qPCR-mediated estimates showed an increase in the proportion of bacteria from HW to AW and KhW (87–96%) for water and from AS to KhS and HS (74–99%) for sediment samples. In comparison, the archaea represent a reverse trend reaching the highest proportion in AS (~ 25%) (Supplementary Fig. S2 and Supplementary Table S1).

### Distinct prokaryotic community composition along the oil pollution continuum of the Persian Gulf

**Figure 2:**
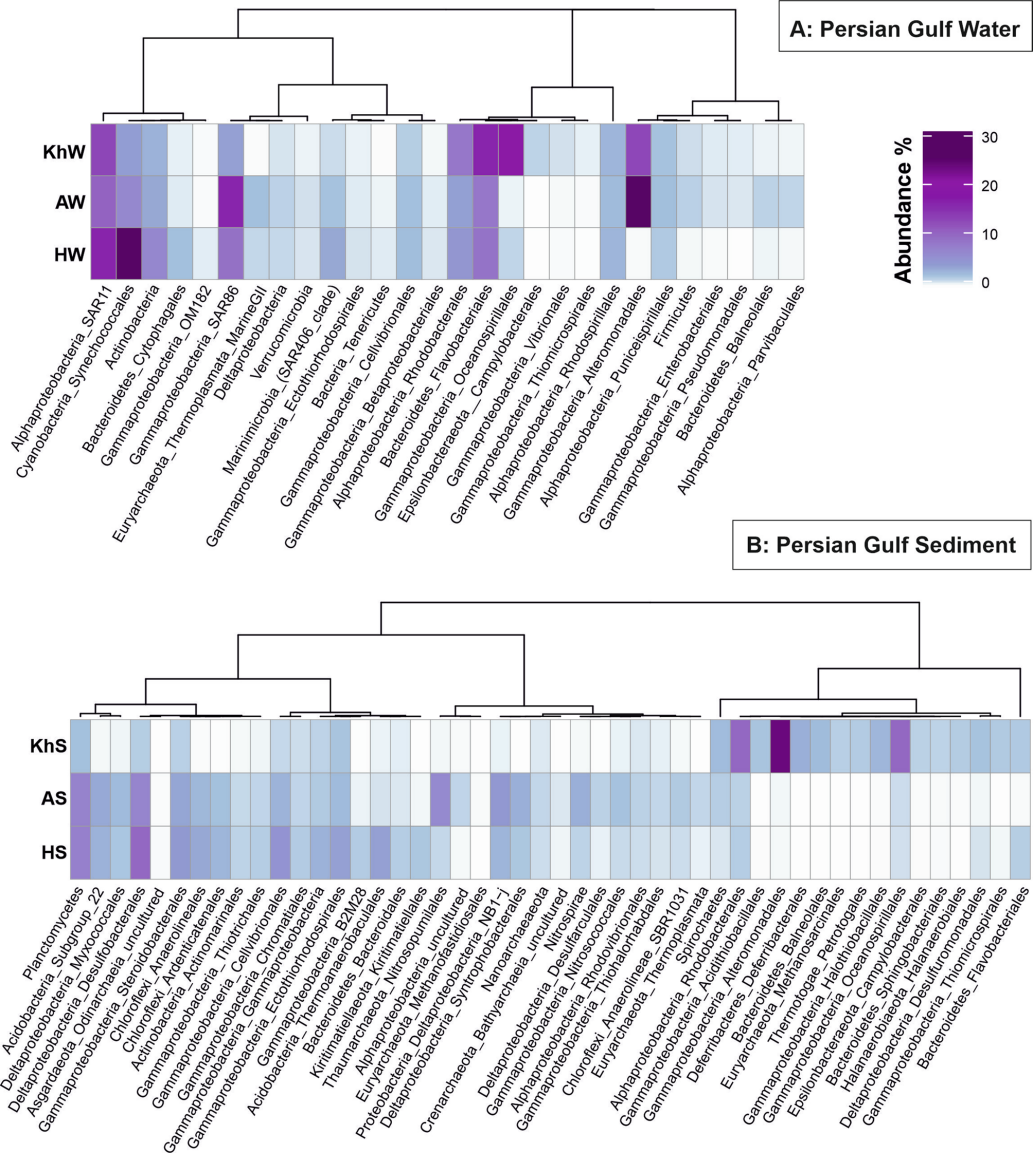
Prokaryotic community composition of the Persian Gulf water (**A**) and sediment (**B**) samples according to the abundance of 16S rDNA reads in unassembled metagenomes. Column names are microbial taxa at the order level. For some taxa with lower frequency, the sum of orders is displayed in their corresponding higher taxonomic level. There are a total number of 28 and 48 taxa for water and sediment samples, respectively by which samples are compared. Rows are the name of samples. Dendrograms represent the clustering of columns based on Pearson correlation. Figure was plotted using “circlize” and “ComplexHeatmap” packages in R.

In pristine marine environments, the internal feedback mechanisms of the microbial communities facilitate keeping a steady “average” composition despite changes imposed by factors such as temperature, nutrient supply, and physical mixing^32,33^. However, in the Persian Gulf, the continuous exposure to oil pollution in water samples causes spatial patchiness and a shift in the microbial community composition resulting in distinct compositions along the pollution continuum. The HW microbial community, which is the input water to the Gulf, had a microbial community similar to what was reported for other marine ecosystems. The community is dominated by *Synechococcales*, SAR11, SAR86, *Flavobacteriales*, *Actinomarinales,* and *Rhodobacterales*. Along the pollution continuum, the community shifted towards a higher relative abundance of *Gammaproteobacteria* (from 13.5 to 48%) and *Bacteroidetes* (from 8 to 16%) representatives (Fig. 2A). The relative abundance of phyla *Cyanobacteria, Actinobacteria, Marinimicrobia,* and order *Cytophagales* of the phylum *Bacteroidetes* decreased; inversely, the relative abundance of phyla *Proteobacteria*, *Epsilonbacteraeota*, and *Firmicutes* as well as orders *Flavobacteriales* and *Balneolales* of the phylum *Bacteroidetes* consistently increased from HW to AW and KhW (Fig. 2A). The order *Synechococcales* negatively responds to oil pollution in marine surface water^34^. Decreased abundance of *Synechococcales* along the oil pollution continuum from HW to AW and KhW complied with our chlorophyll-a measurements (0.24, 0.091, and 0.013 µg/L, respectively) (Supplementary Table S1). This decreased chlorophyll-a concentrations along the pollution continuum highlight the adverse impact of pollution on the Gulf’s primary production. More empirical evidence is needed to unravel the impact of decreased *Synechococcales* abundance and its relation to oil pollution. While oil pollution of the KhW and AW samples was below our detection limit (50 µg/L), their dominant prokaryotic community was remarkably similar to other oil-polluted marine samples^4^. *Oceanospirillales*, *Flavobacteriales*, *Alteromonadales* and *Rhodobacterales* had the highest relative abundance in KhW and the prokaryotic community of AW was mainly comprised of *Alteromonadales*, SAR86, *Flavobacteriales*, *Rhodobacterales,* and *Thermoplasmata* (Fig. 2A).

In response to oil contamination (e.g., in the form of an oil spill), relative abundance of oil-degrading microbes increases (e.g., *Oceanospirillales*, *Flavobacteriales*, *Alteromonadales*, SAR86, and *Rhodobacterales*)^35^. Representatives of SAR86 clade contain cytochrome P450 and dioxygenase genes that are involved in degrading aliphatic and aromatic xenobiotic compounds^36^. They reached the highest relative abundance in AW, where they were exposed to aromatic pollutants. *Alteromonadales* representatives encode enzymes for degrading recalcitrant and toxic branched-chain alkanes, and PAHs thus are mainly involved in the final steps of the degradation process^5^. They showed the highest relative abundance in AW, where most of the oil contaminants were low molecular weight aromatic compounds (Fig. 2A).

*Oceanospirillales* comprised ca. 20% of the KhW prokaryotic community (compared to ca. 1% in HW and AW), suggesting a recent recurring pollution. They prevail following marine oil pollution and are involved in the degradation of labile compounds such as non-branched alkanes and cycloalkanes^37^.

In sediment samples, KhS represented a distinct microbial profile from HS and AS. *Alteromonadales*, *Rhodobacterales*, *Oceanospirillales*, *Deferribacterales*, *Halothiobacillales,* and *Balneolales* (> 2%) representatives were enriched in KhS (Fig. 2B). The co-presence of the orders *Methanosarcinals*, *Alteromanadales,* and *Thermotogae* (*Petrotogales*) in the KhS hints at potential oil reservoir seepage around the sampling site since these taxa are expected to be present in oil reservoirs^38^. The main HC pollutants in the Asalouyeh are low molecular weight aromatic compounds that mainly influence the prokaryotic population in the water column and rarely precipitate into sediments hence the similarity of AS to HS microbial composition as they both experience low pollution rates.

Apart from oil-degrading *Proteobacteria* (e.g. *Alteromonadales*, *Rhodobacterales*, and *Oceanospirillales*), a diversity of sulfur/ammonia-oxidizing chemolithoautotrophic *Proteobacteria* were present in these sediments although at lower abundances e.g., (*Acidithiobacillales* (KhS 1.8%), *Chromatiales* (HS 1.5, AS 1.1, KhS 0.85%), *Ectothiorhodospirales* (HS 3.75, AS 2.3, KhS 1.7%), *Halothiobacillales* (KhS 2.6%), *Thiotrichales* (HS 1.5, AS 1.1, KhS 0.3%), *Thiohalorhabdales* (HS 0.7, AS 1.2, KhS 0.5%), *Thiomicrospirales* (KhS 1.5%)) (Fig. 2B).

Sulfate-reducing bacteria (SRB) in HS comprised up to 16.2% of the community (*Desulfobacterales,* NB1-j, *Myxococcales, Syntrophobacterales,* and *Thermodesulfovibrionia*). Similar groups along with *Desulfarculales,* comprised the SRB functional guild of the AS (~ 18.9%). In comparison, *Desulfuromonadales* and *Desulfobacterales* were the SRB representatives in KhS with a total abundance of only ~ 3.3%. The lower phylogenetic diversity and community contribution of SRBs in KhS hint at the potential susceptibility of some SRBs to oil pollution or that HC degraders might outcompete them (e.g., *Deferribacterales*). Additionally, KhS was gravel-sized sediment (particles ≥ 4 mm diameter), whereas HS and AS samples were silt and sand-sized sediments^39^. The higher oxygen penetration in gravel particles of KhS hampers anaerobic metabolism of sulfate/nitrate-reducing bacteria hence their lower relative abundance in this sample (Fig. 2B).

Whereas in water samples, sulfur/ammonia-oxidizing chemolithoautotrophs such as Thiomicrospirales and sulfate/nitrate-reducing bacteria such as Desulfobacterales, NB1-j, Deferribacterales, Anaerolineales, Nitrosococcales, Nitrosopumilales, and Pirellulales were present in very small quantities (lower than 0.5% in each sample).

### Chronic exposure to oil pollution shapes similar prokaryotic communities as oil spill events

We analyzed the prokaryotic community composition of 41 oil-polluted marine water metagenomes (different depths in the water column) from Norway (Trondheimsfjord), Deepwater Horizon (Gulf of Mexico), the northern part of the Gulf of Mexico (dead zone) and Coal Oil Point of Santa Barbara; together with 65 oil exposed marine sediment metagenomes (beach sand, surface sediments and deep-sea sediments) originating from DWH Sediment (Barataria Bay), Municipal Pensacola Beach (USA) and a hydrothermal vent in Guaymas Basin (Gulf of California) in comparison with the PG water and sediment samples (in total 112 datasets) (Supplementary Table S3). This extensive analysis allowed us to get a comparative overview of the impact of chronic oil pollution on the prokaryotic community composition.

**Figure 3:**
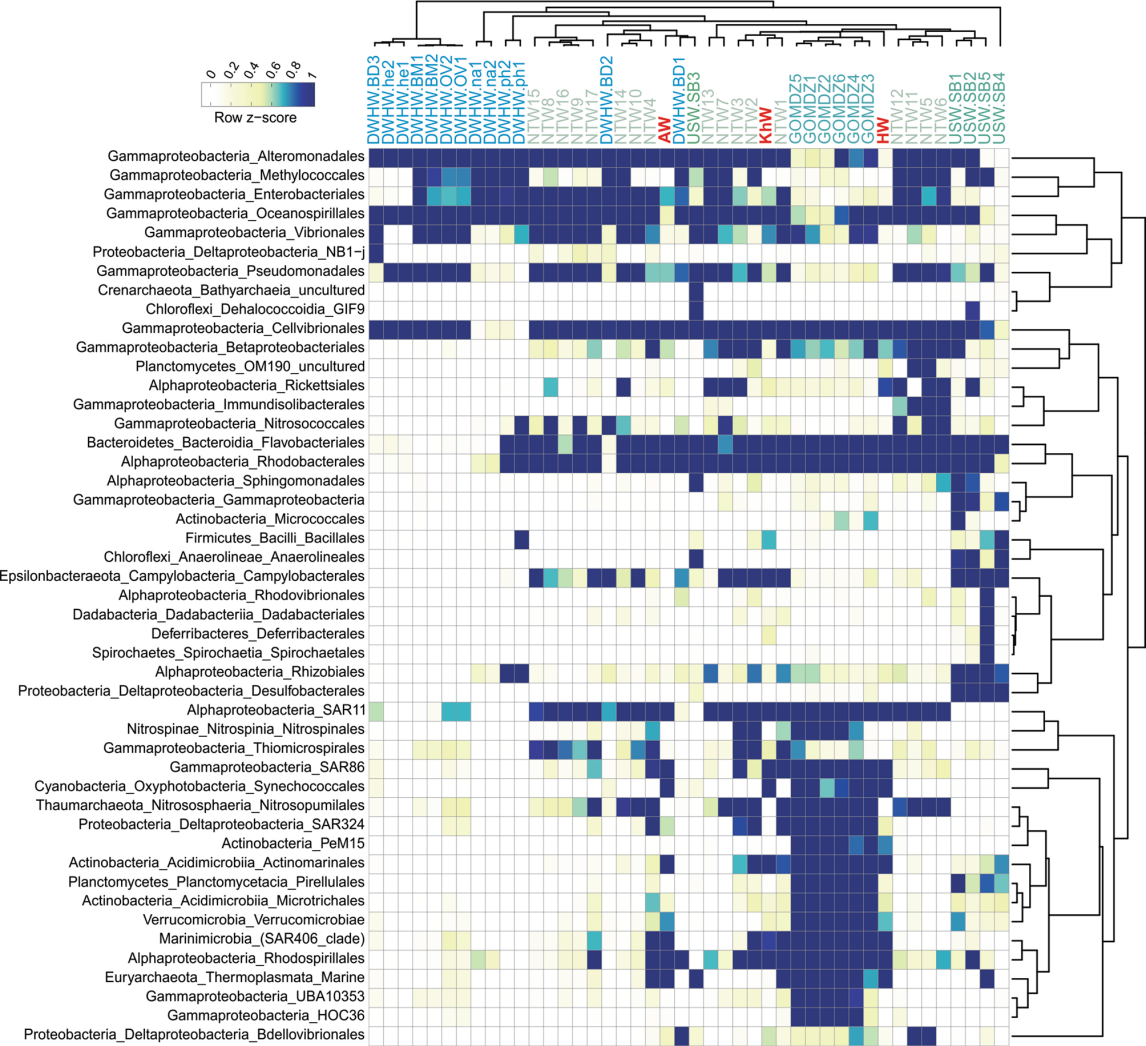
The abundance of unassembled 16S rDNA reads from unassembled metagenomes of different oil-polluted water samples (41). Row names are microbial taxa at the order level. For taxa with lower frequency, the higher taxonomic level is shown (47 taxa in total). The right-hand dendrogram represents the clustering of rows based on the Pearson correlation. Columns are the name of water samples. Samples are clustered based on Pearson correlation and the color scale on the top left represents the row Z-score. Figure was plotted using “circlize” and “ComplexHeatmap” packages in R.

Hydrocarbonoclastic bacteria affiliated to *Oceanospirillales, Cellvibrionales* (*Porticoccaceae* family), and *Alteromonadales*^40^ comprised a significant proportion of the prokaryotic community in samples with higher aliphatic compounds pollution e.g. DWHW.BD3 (sampled six days after the incubation of unpolluted water with Macondo oil), DWHW.he1, and DWHW.he2 (oil-polluted water samples incubated with hexadecane), DWHW.BM1, DWHW.BM2, DWHW.OV1 and DWHW.OV2 (sampled immediately after the oil spill in the Gulf of Mexico) (Fig. 3). Samples treated with Macondo oil, hexadecane, naphthalene, phenanthrene, and those taken immediately after the oil spill in the Gulf of Mexico had a significantly lower proportion of SAR11 due to the dominance of bloom formers and potential susceptibility of SAR11 to oil pollutants (Fig. 3).

*Flavobacteriales* and *Rhodobacterales* were present in relatively high abundance in almost all oil-polluted water samples except for those with recent pollution. Samples named NTW5, NTW6, NTW11, NTW12, which were incubated with MC252 oil for 32–64 days, represented similar prokaryotic composition dominating taxa that are reportedly involved in degrading recalcitrant compounds like PAHs in the middle-to-late stages of the oil degradation process (*Alteromonadales*, *Cellvibrionales*, *Flavobacteriales,* and *Rhodobacterales*). Whereas at the earlier contamination stages, samples represented a different community composition with a higher relative abundance of *Oceanospirillales* (e.g., NTW8, NTW9, NTW15, NTW16, and NTW17 sampled after 0–8 days incubation) (Fig. 3).

**Figure 4:**
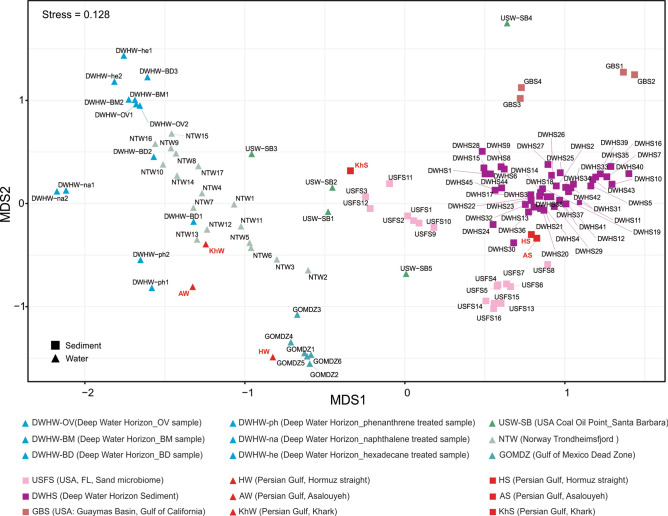
Non-metric multidimensional scaling (NMDS) of the Persian Gulf water and sediment metagenomes along with oil-polluted marine water and sediment metagenomes based on Bray–Curtis dissimilarity of the abundance of 16S rDNA reads in unassembled metagenomes at the order level. Samples with different geographical locations are shown in different colors. PG water and sediment samples are shown in red. Water and sediment samples are displayed by triangle and square shapes, respectively. Figure was plotted using “vegan” library in R.

The non-metric multidimensional analysis of the prokaryotic community of 106 oil-polluted water and sediment samples, together with the PG samples, is represented in Fig. 4. Water and sediment samples expectedly represented distinct community compositions. The AW sample was placed near samples treated with phenanthrene and naphthalene in the NMDS plot showing the impact of aromatic compounds on its microbial community. The KhW sample was located near NTW13 in the plot, both of which had experienced recent oil pollution.

The orders *Oceanospirillales*, *Alteromonadales,* and *Pseudomonadales* were present in relatively high abundances in all oil-polluted water samples except for HW (PG input water) and samples collected from the northern Gulf of Mexico dead zone (GOMDZ) (Fig. 3). Persian Gulf was located in the proximity of the developing oxygen minimum zone (OMZ) of the Arabian Sea that is slowly expanding towards the Gulf of Oman^41^. Potential water exchange with OMZ areas could be the cause of higher similarity to the GOMDZ microbial community^42^.

Our results suggest that water samples with similar contaminants and exposure time to oil pollution enrich for similar phylogenetic diversity in their prokaryotic communities (Fig. 3). Marine prokaryotes represent vertical stratification with discrete community composition across the depth profile. According to our analyses, the prokaryotic communities of the oil-polluted areas are consistently dominated by similar taxa regardless of sampling depth or geographical location. We speculate that the high nutrient input due to crude oil intrusion into the water presumably disturbs this stratification and HC degrading microorganisms are recruited to the polluted sites where their populations flourish.

**Figure 5:**
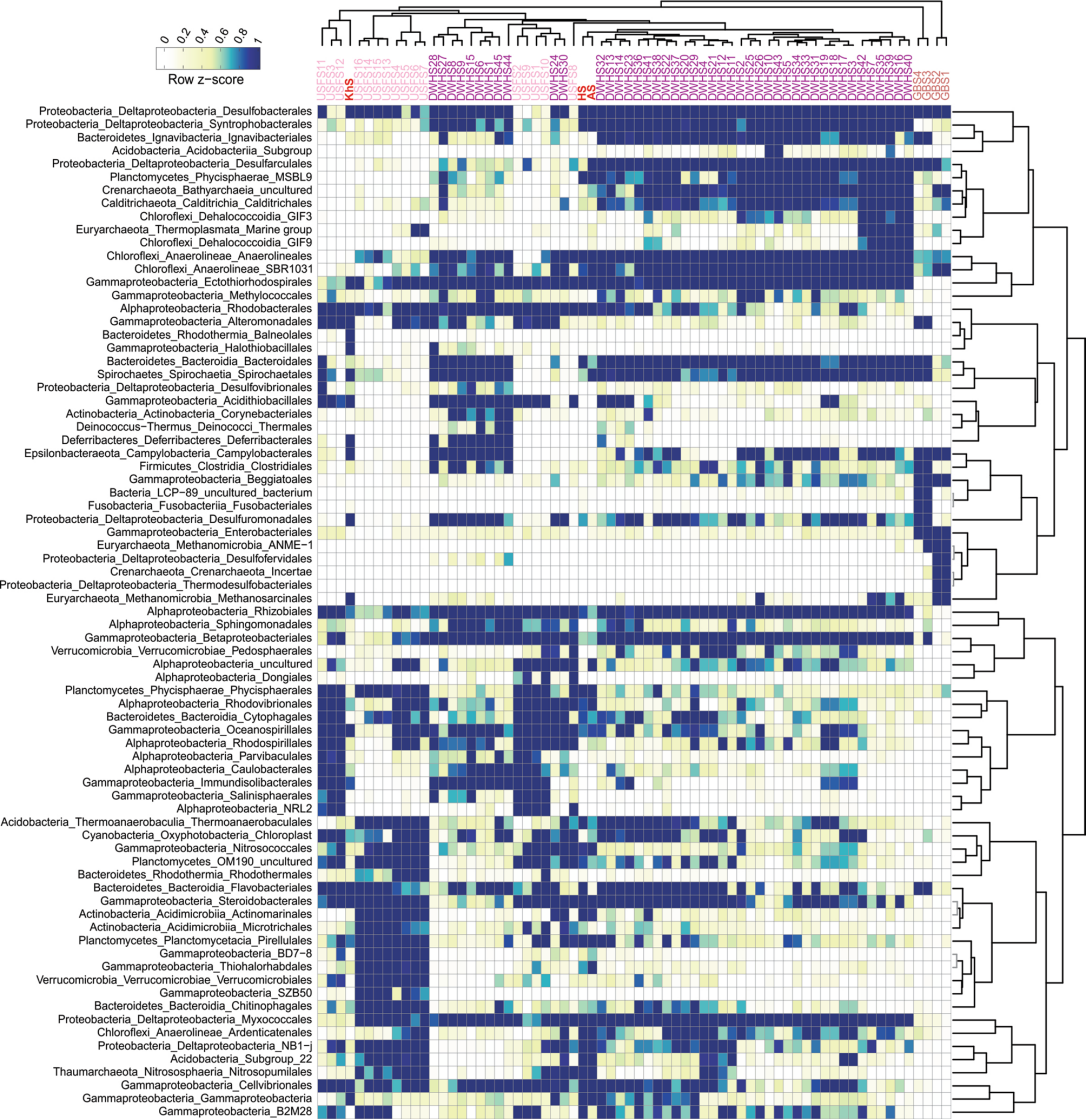
The abundance of unassembled 16S rDNA reads from unassembled metagenomes of different oil-polluted sediment samples (65). Row names are microbial taxa at the order level. For taxa with lower frequency, the higher taxonomic level is shown (77 taxa in total). The right-hand dendrogram represents the clustering of rows based on the Pearson correlation. Columns are the name of sediment samples. Samples are clustered based on Pearson correlation and the color scale on the top left represents the row Z-score. Figure was plotted using “circlize” and “ComplexHeatmap” packages in R.

The inherent heterogeneity of the sediment prokaryotic communities is retained even after exposure to oil pollution, reflected in their higher alpha diversity (Supplementary Fig. S3). However, similar taxa dominate the community in response to oil pollution (Fig. 5).

In sediment samples, *Deltaproteobacteria* had the highest abundance, followed by *Gammaproteobacteria* representatives. *Ectothiorhodospirales, Rhizobiales*, *Desulfobacterales*, *Myxococcales,* and *Betaproteobacteriales* representatives were present in almost all samples at relatively high quantities (Fig. 5). Sulfate/nitrate-reducing bacteria were major HC degraders in sediment, showing substrate specificity for anaerobic HC degradation^43^. *Desulfobacterales* and *Myxococcales* were ubiquitous sulfate-reducers, present in almost all oil-polluted sediment samples^44^. Sulfate-reducing *Deltaproteobacteria* play a key role in anaerobic PAH degradation, especially in sediments containing recalcitrant HC types^45^. Members of *Rhizobiales* are involved in nitrogen fixation, which accelerates the HC removal process in the sediment samples^46^, and therefore their abundance increase in response to oil pollution (Fig. 5).

Prokaryotes involved in nitrogen/sulfur cycling of sediments are defined by factors such as trace element composition, temperature, pressure, and more importantly, depth and oxygen availability. In oil-polluted sediment samples, the simultaneous reduction of available oxygen with an accumulation of recalcitrant HCs along the depth profile complicates the organic matter removal. However, anaerobic sulfate-reducing HC degrading bacteria will cope with this complexity^47^. Prokaryotic communities of HS and AS samples represented similar phylogenetic diversity (Figs. 4, 5). Their prokaryotic community involved in the nitrogen and sulfur cycling resembles the community of DWHS samples. The KhS sample had a similar prokaryotic community to deeper sediment samples collected from 30 to 40 cm depth (USFS3, USFS11, and USFS12) which could be due to our sampling method using a grab sampling device.

Our results show that the polluted sediments’ sampling depth (surface or subsurface) defines the dominant microbial populations. Hydrocarbon degrading microbes had the ubiquitous distribution in almost all oil-polluted water and sediment samples including *Oceanospirillales, Cellvibrionales*, *Alteromonadales*, *Flavobacteriales, Pseudomonadales*, and *Rhodobacterales.* Mentioned orders along with *Ectothiorhodospirales, Rhizobiales*, *Desulfobacterales*, *Myxococcales,* and *Betaproteobacteriales* and also representatives of *Deltaproteobacteria* phylum dominated in sediment samples. However, their order of frequency varies depending on the type of oil pollution present at the sampling location and the exposure time.

### Genome-resolved metabolic analysis of the Persian Gulf’s prokaryotic community along the pollution continuum

A total of 82 metagenome-assembled genomes (MAGs) were reconstructed from six sequenced metagenomes of the PG (completeness ≥ 40% and contamination ≤ 5%). Amongst them, eight MAGs belonged to domain Archaea and 74 to domain bacteria. According to GTDB-tk assigned taxonomy (release89) (https://data.gtdb.ecogenomic.org/releases/release89/), reconstructed MAGs were affiliated to *Gammaproteobacteria* (36.6%), *Alphaproteobacteria* (12.2%), *Flavobacteriaceae* (9.7%), *Thermoplasmatota* (5%) together with some representatives of other phyla (MAG stats in Supplementary Table S4).

**Figure 6:**
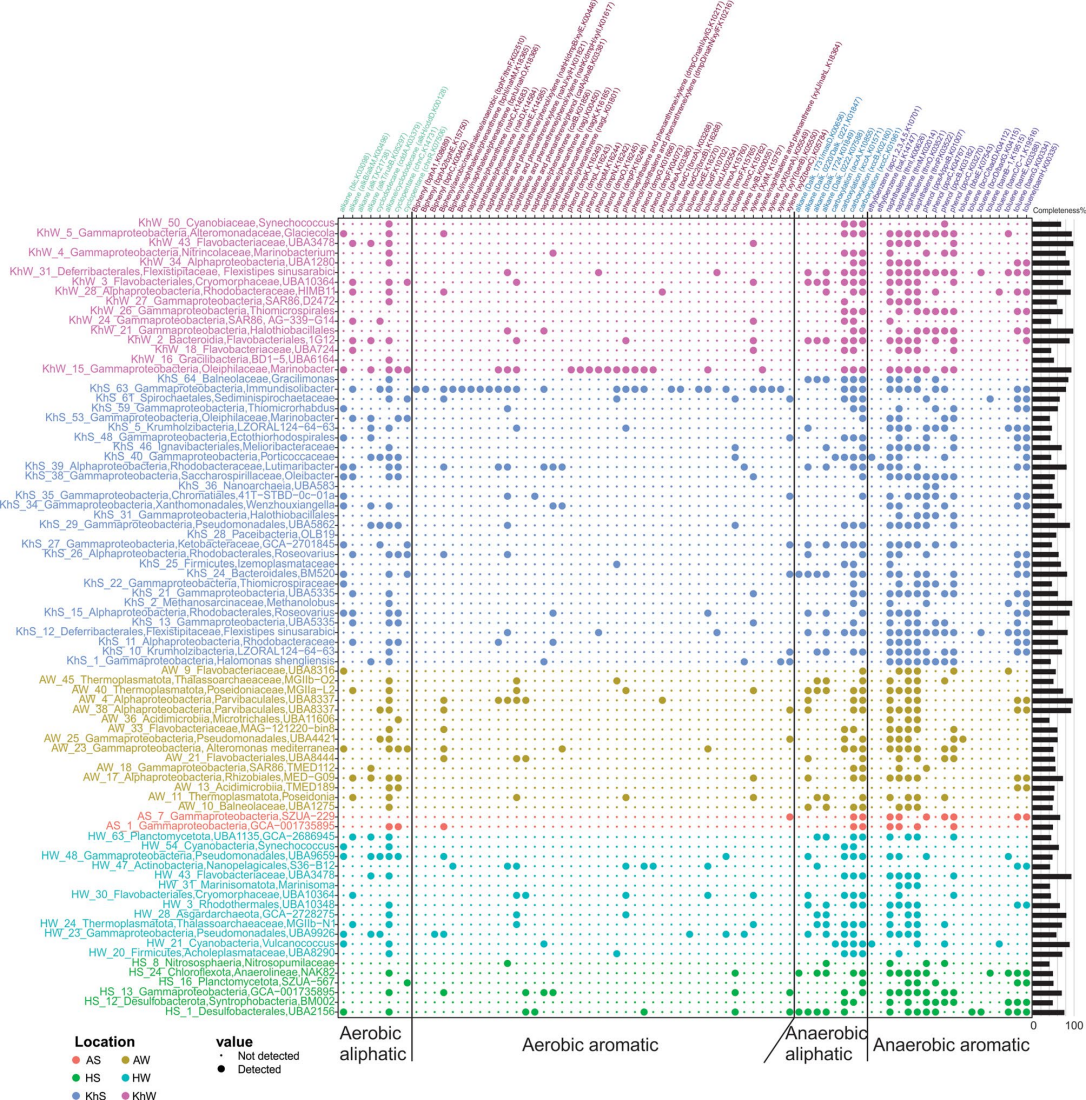
Hydrocarbon degrading enzymes present in recovered MAGs from the PG water and sediment metagenomes. Row names represent the taxonomy of recovered MAGs and their completeness is provided as a bar plot on the right side. The color indicates the MAG origin. The size of dots indicates the presence or absence of each enzyme in each recovered MAG. Columns indicate the type of hydrocarbon and in the parenthesis is the name of the enzyme hydrolyzing this compound followed by its corresponding KEGG orthologous accession number. Figure was plotted using “reshape2” and “ggplot2” packages in R.

A collection of reported enzymes involved in the degradation of different aromatic and aliphatic HCs under both aerobic and anaerobic conditions was surveyed in the annotated MAGs of this study^43,48–50^. The KEGG orthologous accession numbers (KOs) of genes involved in HC degradation were collected, and the distribution of KEGG orthologues detected at least in one MAG (n = 76 genes) is represented in Fig. 6.

A combination of different enzymes runs the oil degradation process. Mono- or dioxygenases are the main enzymes triggering the HC degradation process under aerobic conditions. Under anaerobic conditions, degradation is mainly started by the addition of fumarate or in some cases, by carboxylation of the substrate. Therefore, bacteria containing these genes will potentially initiate the degradation process that will be continued by other heterotrophs. Enzymes such as decarboxylase, hydroxylase, dehydrogenase, hydratase, and isomerases act on the products of initiating enzymes mentioned above through a series of oxidation/reduction reactions.

Various microorganisms cooperate to cleave HCs into simpler compounds that could enter common metabolic pathways. Mono- or dioxygenases which are involved in the degradation of alkane (alkane 1-monooxygenase, alkB/alkM), cyclododecane (cyclododecanone monooxygenase, cddA), Biphenyl (Biphenyl 2, 3-dioxygenase subunit alpha/beta, bphA1/A2, Biphenyl-2, 3-diol 1, 2-dioxygenase, bphC), phenol (phenol 2-monooxygenase, pheA), toluene (benzene 1, 2-dioxygenase subunit alpha/beta todC1/C2, hydroxylase component of toluene-4-monooxygenase, todE), xylene (toluate/benzoate 1,2-dioxygenase subunit alpha/beta/electron transport component, xylX/Y/Z, hydroxylase component of xylene monooxygenase, xylM) and naphthalene/phenanthrene (catechol 1,2 dioxygenase, catA, a shared enzyme between naphthalene/phenanthrene /phenol degradation) were detected in recovered MAGs of the PG.

The key enzymes including Alkylsuccinate synthase (I)/(II) (assA1/A2), benzylsuccinate synthase (BssA)/benzoyl-CoA reductase (BcrA), ethylbenzene dehydrogenase (EbdA), and 6-oxo-cyclohex-1-ene-carbonyl-CoA hydrolase (BamA) that are responsible for initiating the degradation of alkane, toluene, ethylbenzene and benzoate exclusively under anaerobic conditions were not detected in reconstructed MAGs of this study. Consequently, recovered MAGs of this study are not initiating anaerobic degradation via known pathways while they have the necessary genes to continue the degradation process started by other microorganisms.

The MAG KhS_63 affiliated to *Immundisolibacter* contained various types of mono- or dioxygenases and had the potential to degrade a diverse range of HCs such as alkane, cyclododecane, toluene, and xylene (Fig. 6). Members of this genus have been reported to degrade high molecular weight PAHs^51^.

*Lutimaribacter* representatives have been isolated from seawater and reported to be capable of degrading cyclohexylacetate^52^. We also detected enzymes responsible for alkane, cycloalkane (even monooxygenase enzymes), and naphthalene degradation under aerobic conditions and alkane, ethylbenzene, toluene, and naphthalene degradation under anaerobic conditions in KhS_39 affiliated to this genus (Fig. 6).

The MAGs KhS_15 and KhS_26 affiliated to *Roseovarius* had the enzymes for degrading alkane (alkane monooxygenase, aldehyde dehydrogenase), cycloalkane, naphthalene, and phenanthrene under aerobic and toluene and naphthalene under anaerobic condition. PAHs degradation has been reported for other representatives of this taxa as well^53^.

The MAGs KhS_11 (a representative of *Rhodobacteraceae*) and KhS_53 (*Marinobacter*) had alkB/alkM, KhS_27 (GCA-2701845), KhS_29 (UBA5862) and KhS_40 (from *Porticoccaceae* family) had cddA, KhS_13 and KhS_21 (UBA5335) and KhS_38 (*Oleibacter*) had both alkB/alkM and xylM genes. They were among microbes that were initiating the degradation of alkane, cycloalkane and xylene compounds. Other MAGs recovered from Khark sediment were involved in the continuation of the degradation pathway. For example, KhS_1 was affiliated to the genus *Halomonas* and had different enzymes to degrade intermediate compounds. *Halomonas* representatives have been frequently isolated from oil-polluted environments^54^. The phylum *Krumholzibacteria* has been first introduced in 2019 and reported to contain heterotrophic nitrite reducers^55^. Two MAGs, KhS_5 and KhS_10, were affiliated to this phylum and contained enzymes involved in the anaerobic degradation of toluene, phenol, and naphthalene (Fig. 6).

The MAGs KhS_12 and KhW_31 affiliated to the genus *Flexistipes*, in *Deferribacterales* order, were reconstructed from both KhW and KhS samples. *Deferribacterales* are reported to be present in the medium to high-temperature oil reservoirs with HC degradation activity and also in high-temperature oil-degrading consortia^56^. The type strain of this species was isolated from environments with a minimum salinity of 3% and a temperature of 45–50 °C^57^. The presence of this genus in KhS could be due to natural oil seepage from the seabed as PG reservoirs mainly have medium to high temperature and high salinity. Enzymes involved in the degradation of alkane, phenol, toluene and naphthalene under anaerobic conditions were present in MAGs KhS_12 and KhW_31.

As mentioned earlier, *Flavobacteriales* are potent marine indigenous HC degraders that bloom in response to oil pollution^58^. *Flavobacteriales* affiliated MAGs (KhW_2, KhW_3, AW_21, and AW_33) were recovered from KhW and AW and mostly contained enzymes that participate in the degradation of aromatic compounds under anaerobic conditions. KhW_2 and KhW_3 also had both alkB/M (alkane monooxygenase) and xylM enzyme, which initiates the alkane and xylene bioremediation in Khark water. Among other recovered MAGs from KhW sample, KhW_18 (UBA724), KhW_24 (clade SAR86), KhW_43 (UBA3478) had alkB/M, and xylM, KhW_24 (clade SAR86) had alkB/M and cddA, and KhW_28 (from *Rhodobacteraceae* family) had alkB/M and pheA genes in their genome to initiate the degradation process (Fig. 6).

*Marinobacter* (KhW_15) was another MAG reconstructed from KhW sample. This genus is one of the main cultivable genera that play a crucial role in the bioremediation of a wide range of oil derivatives in polluted marine ecosystems^54^.

Marine Group II (MGII) and *Poseidonia* representatives of *Thermoplasmatota* that have been reported to be nitrate-reducing *Archaea*^59^, were recovered from AW sample (AW_40, AW_45) and contained several enzymes contributing in alkane (alkane monooxygenase, aldehyde dehydrogenase) and naphthalene/phenanthrene/phenol/xylene degradation (decarboxylase) under aerobic conditions. The HC degradation potential of representatives of this phylum has been previously reported^60^.

In the Asalouyeh water sample, MAGs AW_25 (UBA4421) and AW_38 (UBA8337) had cddA, AW_21 (UBA8444) had catA, AW_11 (*Poseidonia*) and AW_17 (from *Rhizobiales* order) had both alkB/M and xylM, and AW_4 (UBA8337) had catA and pheA genes and had potential to trigger the breakdown of their corresponding oil derivatives.

Other recovered genomes had the potential to metabolize the product of initiating enzymes. For instance, AW_23 contained enzymes involved in the degradation of naphthalene, phenol and cyclododecane and was affiliated to the genus *Alteromonas* (Fig. 6).

Three recovered MAGs of HW affiliated to *Pseudomonadales* (HW_23), *Poseidoniales* (HW_24), and *Flavobacteriales* (HW_30) contained some initiating enzymes to degrade cyclododecane/biphenyl/toluene, alkane/xylene, and alkane/xylene/naphthalene/phenanthrene, respectively. A representative of *Heimdallarchaeia* that are mainly recovered from sediment samples was reconstructed from the Hormuz water sample (HW_28). It had a completeness of 81% and contained enzymes involved in anaerobic degradation of alkanes. This archaeon could potentially be an input from the neighboring OMZ as this phyla include representatives adopted to microoxic niches^61^. Containing genes with the potential to initiate the oil derivative degradation in the input water with no oil exposure reiterates the intrinsic ability of marine microbiota for HC degradations and oil bioremediation.

While 16S rRNA provides an overview of the community, MAGs provide the possibility to inspect the metabolic capability of the microbiota. We decided to provide both in this manuscript as we believe they are complementary. Having the full picture provided by the combination of these analyses allows for a better understanding of the community structure and their metabolic capabilities. This is even more evident for sediment samples as they are highly diverse, and reconstructing MAGs from sediment metagenomes is still a bottlenecks. In this case, we rely more on the 16S rRNA to provide an overall view of the community composition.

This said, we see similar taxonomic distribution in the MAGs and 16S rRNA e.g., the prevalence of Flavobacteriales and Rhodobacterales in KhW and KhS, Synechococcales, and Desulfobacteriales and Flavobacteriales in HW, HS and AW samples, respectively.

Additionally, some rare microbiota representatives were recovered among reconstructed MAGs. For example, the Immundisolibacterales showed an abundance of only 0.8% in the KhS sample based on 16S rRNA but the recovered KhS_63 MAG was affiliated to this taxon. Notably, this MAG contained many genes involved in hydrocarbon degradation having the highest potential in hydrocarbon degradation.

## Conclusion

Exploring the marine microbial communities’ response to oil pollutions has received increasing attention over the last decade, specifically after the “Deepwater Horizon oil spill”. However, the influence of long-term exposure to oil derivatives in ecosystems such as the Persian Gulf that hosts almost half of the world’s oil reserves and has been chronically exposed to recurrent natural and accidental oil pollutions has remained entirely unknown. Understanding the microbial dynamics in response to oil pollution at different locations of the Persian Gulf can function as a valuable model system for advancing our knowledge and preparedness for managing oil spill accidents in the future.

**Figure 7:**
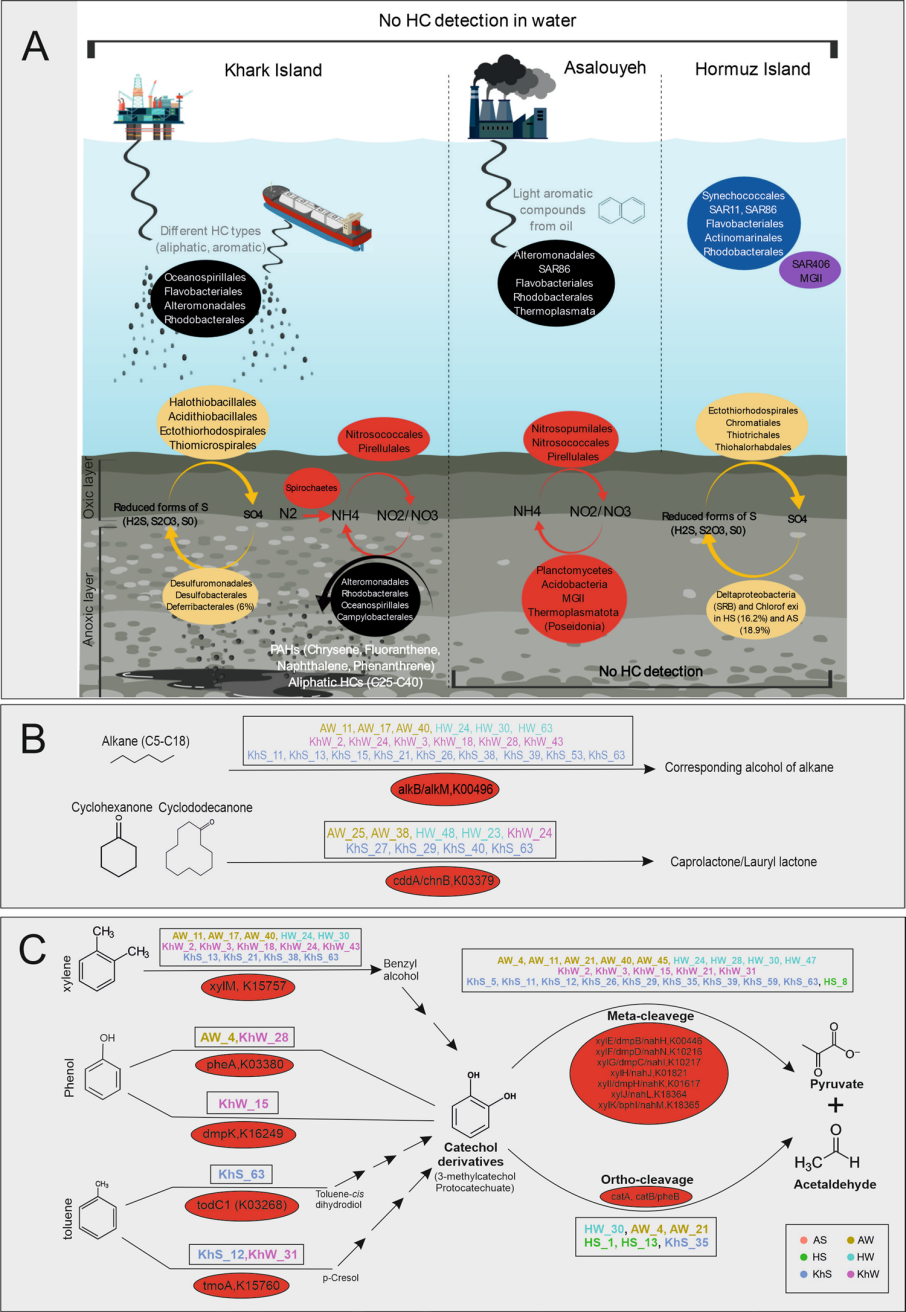
The microbial community dynamics of the Persian Gulf water and sediment samples in response to oil pollution and their degradation potential. (**A**) Overview based on16S rDNA abundance. Hormuz Island was considered as a control location with the least impact from oil pollution. Taxa written in the blue frame are prevalent marine representatives present in HW. Microbial taxa in the Purple frame are mainly detected in OMZ areas and are also present in HW. Samples collected from Asalouyeh province are exposed to potential pollution caused by Gas field wastes. High oil trafficking, oil exploration and extraction, and natural oil seepage are the primary potential pollution sources in Khark Island. The possible pollutant types are shown in gray; however, the hydrocarbon pollution was below the detection limit in collected water samples. Black circles represent microorganisms that are involved in HC degradation in water samples from Asalouyeh and Khark Island. Microbes involved in sulfur and nitrogen cycle are shown in yellow and Red circles, respectively. HS and AS had similar silt and sand-sized sediments with HC below the detection limit. KhS had gravel-sized particles and showed the highest oil pollution shown in white. (**B**) MAGs containing key enzymes for degradation of aliphatic and cycloalkane compounds under aerobic conditions. (**C**) MAGs containing key enzymes for degradation of aromatic compounds under aerobic conditions. PG MAGs did not have Key enzymes for hydrocarbon degradation under anaerobic conditions therefore, it is not shown. Red circles in B and C panels represent key enzymes involved in the degradation. The name of MAGs containing mentioned enzymes are written in rectangles. The MAGs are colored based on the samples they have been recovered from and the legend is shown in the lower right corner. Figure has been created with “BioRender.com”.

Our extensive analysis of available oil-polluted water and sediment metagenomes (*n* = 106), together with the Persian Gulf samples (*n* = 6), showed that the chronic exposure to trace amounts of oil derivatives had altered the microbial community of the Persian Gulf. Even though the pollution remained below our detection limit of 50 µg/L, the long-standing trace oil pollution imposed a consistent selection pressure on the microbial community of the input water, selecting for oil-degrading microbes capable of degrading major locally enriched pollutants (Fig. 7).

Our results showed that regardless of the water column depth and initial composition, the microbial community converges in response to the pollution. It seems that microbes capable of degrading more labile components of the pollutant will be recruited to the pollution zone. Their population will experience a bloom, which will be followed by the next populations capable of degrading more recalcitrant components. Our genome-resolved analyses showed that these microbes employ an intricate “division of labor” to initiate and carry out different stages of the bioremediation process. Higher-resolution spatiotemporal analysis of the microbial community of this highly heterogeneous ecosystem in future studies can reveal critical ecological adaptations to oil pollutants.

## Supplementary Information

Supplementary Table S1.

Supplementary Table S1.

Supplementary Figures.

Supplementary Table S2.

Supplementary Table S3.

Supplementary Table S4.

Supplementary Table S5.

## Data Availability

The metagenomic Raw read files of the Persian Gulf water and sediment samples and all the metagenome-assembled genomes (MAGs) reconstructed (Supplementary Table S5) in this study are archived at the DDBJ/EMBL/GenBank and can be accessed under the Bioproject PRJNA575141.

## Abbreviations

PG: Persian Gulf
HC: Hydrocarbon
PAHs: Polyaromatic hydrocarbons
