# Supplementary material for: Distinct microbial community along the chronic oil pollution continuum of the Persian Gulf converge with oil spill accidents
Source: Sci Rep. 2021 May 31;11:11316. doi: 10.1038/s41598-021-90735-0 (PMC8166890; doi:10.1038/s41598-021-90735-0)
Supplement: Supplementary file 3 — Supplementary Figures. [file 41598_2021_90735_MOESM3_ESM.pdf]

# Supplementary Figures

1

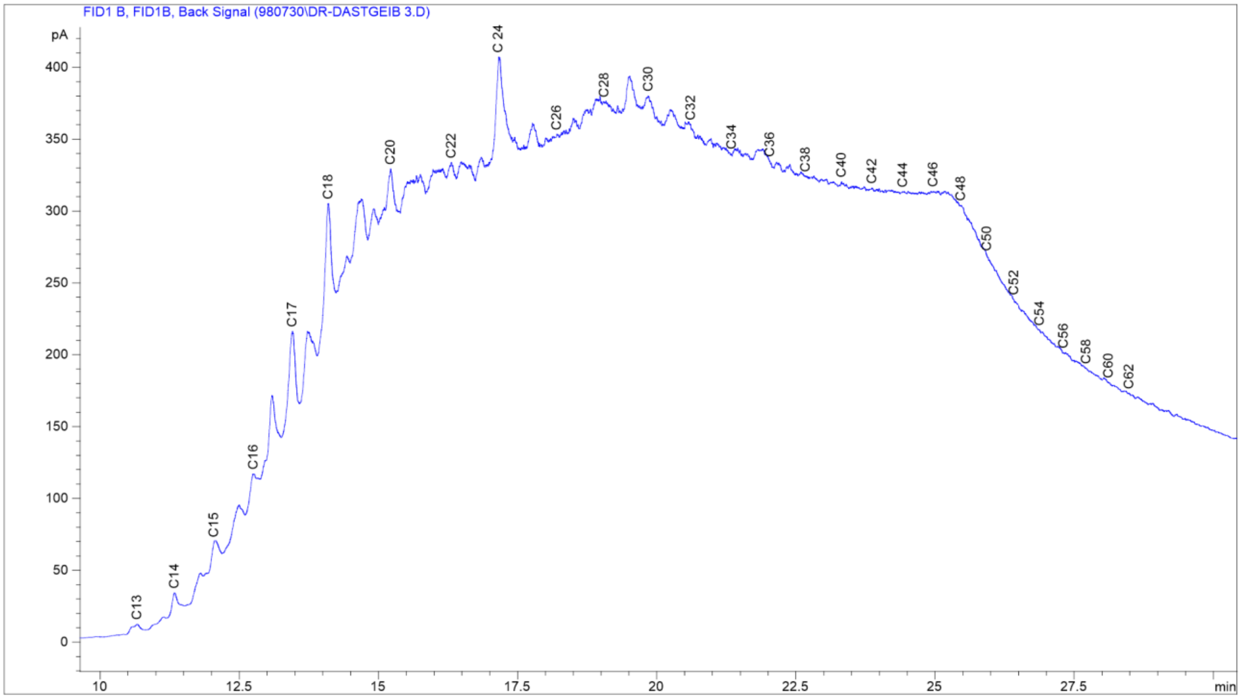


**Supplementary Figure S1.** The carbon distribution of the bulk hydrocarbon compounds extracted from KhS sample measured by GC-SimDis method.

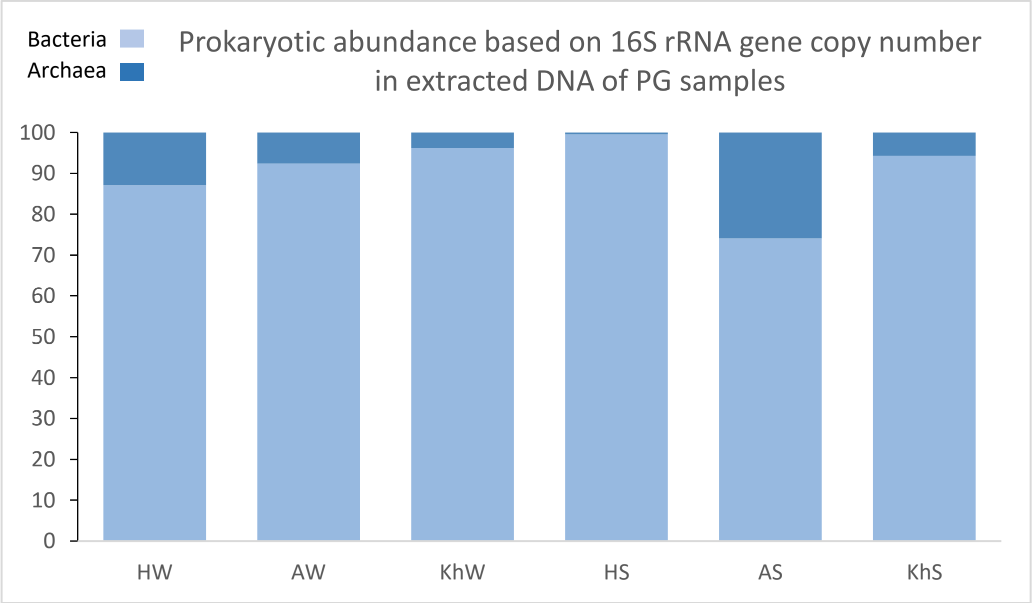


**Supplementary Figure S2.** Prokaryotic 16S rRNA gene copy number abundance measured by qPCR presented as percentage.

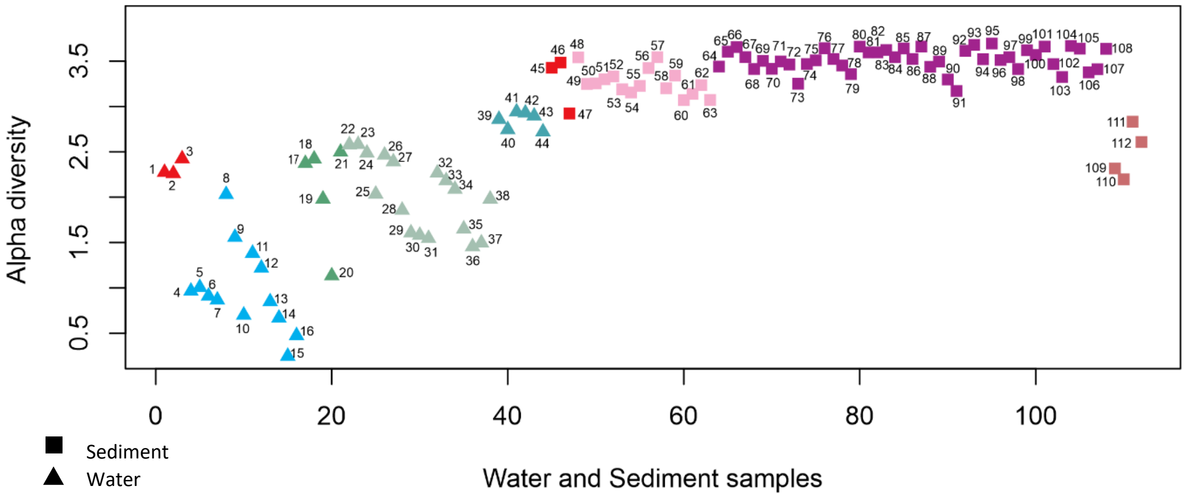


| | | | |
|---|---|---|---|
| [illegible] | [illegible] | [illegible] | [illegible] |
| 9- DWHW-BD2 | 10- DWHW-BD3 | 11- DWHW-ph1 | 12- DWHW-ph2 |
| 13- DWHW-na1 | 14- DWHW-na2 | 15- DWHW-he1 | 16- DWHW-he2 |
| 17-21: USW-SB1- USW-SB5 | 22-38: NTW1-NTW17 | 39-44: GOMDZ1-GOMDZ6 | 45- HS |
| 46- AS | 47- KhS | 48-63: USFS1-USFS16 | 64-110: DWHS1-DWHS45 |
| 111-114: GBS1-GBS4 | | | |

**Supplementary Figure S3.** Alpha diversity of oil-polluted marine water and sediment samples together with the water and sediment samples collected from the Persian Gulf based on Shannon-Wiener index of the abundance of 16S rRNA gene in the unassembled reads clustered in the order level. Samples are color-coded as figure 1. Water and sediment samples are displayed by triangle and square shapes respectively.
